# A Self-Adaptive Strip Pooling Network for Segmenting the Kidney Glomerular Basement Membrane

**DOI:** 10.3390/s25061829

**Published:** 2025-03-14

**Authors:** Caifang Song, Xiangsheng Huang, Xiangyu Lyu

**Affiliations:** 1 School of Mathematics and Computer Science, Shanxi Normal University, Taiyuan 030031, China; lvxiangyu11@gmail.com; 2 Hebei Key Laboratory of Cognitive Intelligence, Xiong’an Institute of Innovation, Xiong’an New Area 071700, China; huangxiangsheng@xii.ac.cn

**Keywords:** self-adaptive strip pooling, attention mechanism, glomerular basement membrane (GBM), skeleton extraction

## Abstract

Accurate semantic segmentation and automatic thickness measurement of the glomerular basement membrane (GBM) can aid pathologists in carrying out subsequent pathological diagnoses. The GBM has a complex ultrastructure and irregular shape, which makes it difficult to segment accurately. We found that the shape of the GBM is striped, so we proposed an RSP model to extract both the strip and square features of the GBM. Additionally, grayscale images of the GBM are similar to those of surrounding tissues, and the contrast is low. We added an edge attention mechanism to further improve the quality of segmentation. Moreover, we revised the pixel-level loss function to consider the tissues around the GBM and locate the GBM as a doctor would, i.e., by using the tissues as the reference object. Ablation experiments with each module showed that SSPNet can better segment the GBM. The proposed method was also compared with the existing medical semantic segmentation model. The experimental results showed that the proposed method can obtain high-precision segmentation results for the GBM and completely segment the target. Finally, the thickness of the GBM was calculated using a skeleton extraction method to provide quantitative data for expert diagnosis.

## 1. Introduction

In 2002, the National Kidney Foundation (NKF) formulated a concise definition of chronic kidney disease (CKD) and standardized the diagnostic criteria, thereby facilitating extensive epidemiological studies targeting the general population. This move enabled researchers to gain a more comprehensive understanding of the prevalence, progression, and associated factors of CKD [1]. A large number of studies on people with kidney disease have been carried out around the world. The results of these studies showed that the incidence of kidney disease is approximately 10%, while only approximately 12% of these people know that they are sick, and that an imbalance between the number of relevant pathologists and patients leads to inefficient diagnoses [2]. CKD has become an important disease that threatens global public health. Kidney puncture biopsy pathology is an important means for diagnosing kidney disease. Transmission electron microscopy is an effective technique for CKD screening. Using the advanced technology of transmission electron microscopy, healthcare professionals can gain a deeper understanding of the intricate, submicroscopic changes that occur within glomerular cells in pathological conditions. This heightened level of detail allows for a more thorough and accurate pathological diagnosis, empowering doctors to make more informed treatment decisions and ultimately improve patient outcomes.

**Figure 1:**
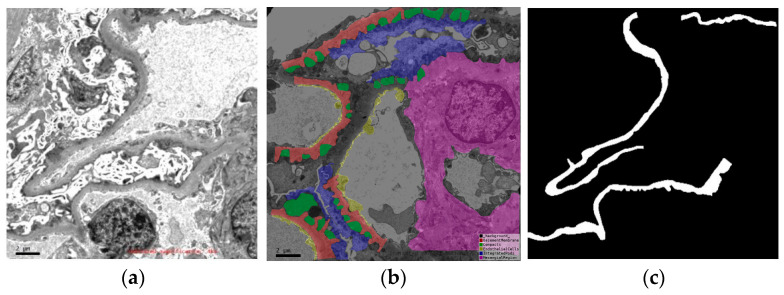
An electron microscopic image derived from a renal biopsy. (**a**) The original electron microscopic image. (**b**) A comprehensively annotated version, where various components are highlighted in distinct colors: the glomerular basement membrane (GBM) in red, compacts in green, endothelial cells in yellow, the mesangial region in blue, and podocytes in purple. (**c**) The GBM, with the image showcasing a refined marking of this critical structure alone.

It can be observed in Figure 1 that the gray image texture corresponding to the glomerular ultrastructure is complex, so it is difficult to identify and judge pathological changes. It is very difficult to observe such complicated pathological images and make diagnoses with the naked eye for an extended period. In the submicroscopic structure of glomerular cells, changes in the glomerular basement membrane (GBM) are closely related to chronic kidney disease (CKD). Doctors often need to identify and measure the GBM in the process of pathological diagnosis. Most of the GBM has low contrast with the surrounding tissue structures. It is not only difficult but also time-consuming to identify changes with the naked eye. Employing computer technology to aid pathologists in the precise and automated identification of the GBM holds immense potential for mitigating diagnostic challenges and enhancing both the efficiency and precision of pathological assessments. This technological advancement stands to revolutionize the field by streamlining the diagnostic process and ensuring a higher degree of accuracy, ultimately benefiting patients through more informed treatment decisions.

In recent years, deep learning techniques have undergone remarkable advancements in the domain of multi-structural medical image segmentation, marking a significant milestone in enhancing the precision and efficiency of this complex task. In this paper, based on the striping features of the GBM, strip pooling and standard pooling were used to extract the texture of the GBM. At the same time, the grayscale of the GBM is similar to that of surrounding tissues, thus leading to low contrast. To further elevate the quality of segmentation outcomes, this paper incorporated an edge attention mechanism, a strategic enhancement aimed at refining the delineation of structural boundaries within medical images. Finally, we revised the pixel-level loss function to consider tissues around the GBM and located the GBM like a doctor would by taking the tissue as the reference object.

## 2. Related Work

Early studies on the automatic semantic segmentation of the GBM were focused on traditional, artificially designed grayscale, morphological, and textural features. As far back as 1993, Ong et al. [3] introduced the groundbreaking use of self-adaptive window-based tracking techniques for segmenting glomerular electron microscopy images, setting a precedent in the field. From then on, some semi- and full-automatic methods have been proposed. By employing the advanced method of region segmentation coupled with dynamic contour modeling, Kamenesky [4] and Rangayyan et al. [5] were able to effectively segment and quantify the GBM, achieving precise measurements and insights. Wu et al. [6,7] presented two methods: The first involves obtaining the centerline of the GBM by interpolating manually marked points, followed by automatic segmentation of the GBM using distance mapping and low-pass filtering techniques. The other method uses a threshold method and a morphological method without the involvement of artificial markers. In addition to segmenting the GBM, Liu et al. [8] also measured its length and calculated the number of gaps present. In the past two years, new segmentation methods, such as image block matching [9] and the use of particle filters [10], have been gradually introduced. In 2017, Guo et al. [11] introduced a novel algorithm based on neutrosophic sets, and shearlet transform has been proposed to enhance the accuracy of glomerular basement membrane segmentation. This approach effectively improves segmentation precision and efficiency by integrating features extracted through the shearlet transform with neutrosophic image processing. In 2019, Cao et al. used random forests to segment the GBM. This method achieved good results, but it is not good enough for medical image segmentation because the false positive rate is high [12]. While the aforementioned methods have indeed contributed to the field, numerous challenges remain unaddressed. These approaches often rely heavily on manual initialization, which not only imposes an additional burden on pathologists but also introduces potential subjective biases. Additionally, they are typically limited to segmenting truncated GBM fragments, focusing primarily on contrast enhancement and unidirectionality. Consequently, ensuring high-quality segmentation of complex GBM images in their entirety remains an ongoing challenge.

In recent years, multi-structure medical image segmentation has witnessed significant advancements driven by deep learning methods. Lei et al. conducted a comprehensive survey exploring the various applications of deep learning techniques in medical image segmentation [13,14]. Most existing medical images are clustered, such as images of the pancreas, retina, brain tumors, and bone marrow. However, the GBM is striped. Current techniques employed for semantic segmentation in medical imaging are not readily transferable to the specific task of GBM segmentation, necessitating alternative or adapted strategies. In 2020, Qi bin Hou et al. [15] introduced a strip pooling approach that markedly improves the capture of long-range dependencies. We further refined this method and integrated it into existing medical image semantic segmentation frameworks, thereby enhancing the segmentation of intricate strip texture features. The new model, called self-adaptive strip pooling (SSP), automatically extracts both the strip and square features of the GBM.

## 3. SSPNet Method

Semantic segmentation models are usually encoding–decoding frameworks. The input of the encoder is downsampled to concentrate information, while the decoder is responsible for upsampling to restore the input size. In the context of semantic segmentation, the encoder plays a pivotal role in extracting crucial contextual information about objects, necessitating a robust multiscale capability. The Res2Net framework achieves this by incorporating hierarchical residual-like connections within a single residual block. This approach enables the model to represent features across multiple scales at a granular level, thereby enhancing the receptive field range for each network layer [16]. Therefore, we used Res2Net as the base model in this paper. To elaborate further, for a given input image, our approach involves extracting five distinct levels of features, denoted as f_i_, where i ranges from 1 to 5, utilizing a Res2Net-based backbone network. Subsequently, we categorized these features into two groups: low-level features, comprising f_1_ and f_2_, and high-level features, which encompass f_3_, f_4_, and f_5_.

**Figure 2:**
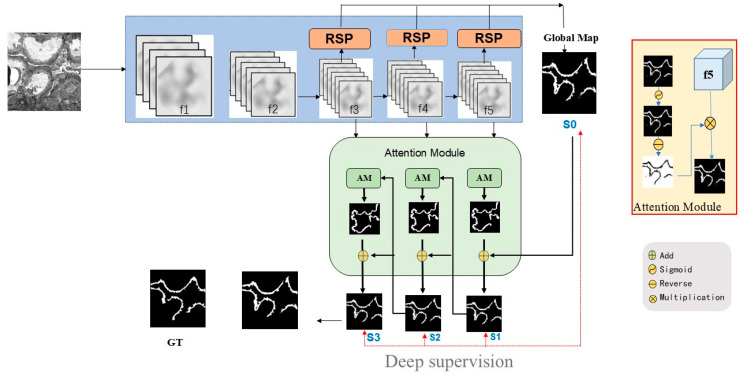
Overview of the proposed SSPNet. The backbone is Res2Net. Due to dilated convolution, f3, f4, and f5 are the same size. We added an RSP module to extract both strip and square features. A set of attention modules were added to enhance the details. We employed deep supervision for three side outputs (S1, S2, S3) and the global map (S0), as indicated by the red dotted line, to ensure optimal performance across all stages of the model.

For the decoder, a loss of information due to downsampling is crucial for segmentation tasks. A bilinear upsampling process usually fails to restore information loss. To further enhance the Res2Net network, we integrated dilated convolutions, facilitating exponential expansion of the receptive field while maintaining resolution and coverage. Leveraging the strengths of Res2Net, we introduced a novel deep neural network architecture, called the self-adaptive strip pooling network (SSPNet), for the GBM segmentation task. As shown in Figure 2, the sizes of f_3_, f_4_, and f_5_ are the same because of the use of dilated convolution.

First, we observed that the shape of the GBM exhibits a striped pattern. Recognizing the limitation of traditional pooling methods in adequately capturing such strip information, we devised and incorporated a refined strip pooling (RSP) module into our framework. This RSP module was specifically designed to extract and integrate the mixed information embedded within the striped features, thereby enhancing the overall feature representation capabilities of our model.

Second, grayscale images of the GBM are similar to grayscale images of surrounding tissues, and the contrast is low. Therefore, an attention module was added to strengthen the edge information.

Finally, in order to mimic the expertise of doctors in locating the GBM while considering the surrounding tissue, we introduced a novel loss function. This function treats the tissue as a reference object, ensuring that the GBM is identified in a manner similar to that of a medical professional approaching the task.

We integrated attention mechanisms and a novel loss function. These mechanisms enable the network to automatically learn and adjust the importance of different features, thereby achieving self-adaptation and optimization of feature representations. Consequently, our network was named the self-adaptive strip pooling network (SSPNet). The details of each component are explained in the following sections.

### 3.1. Self-Adaptive Strip Pooling Module

While the semantic segmentation method, rooted in the fully convolutional network (FCN), has proven adept at capturing high-level semantic information, it predominantly relies on local convolution and pooling operations, resulting in a constrained receptive field. This limitation hampers its effectiveness in analyzing complex scenes, leaving room for improvement. Researchers may use attention mechanisms to model long-distance dependencies [17,18]. Alternative strategies to address the issue of a limited receptive field in semantic segmentation methods include utilizing dilated convolutions [19,20,21], which broaden the receptive field without introducing additional parameters, or employing global or pyramid pooling techniques [22] to enrich global contextual cues. However, the limitation of these methods is that they are mainly used when the input feature is square, and the effect is not very good for many long-strip targets.

In certain scenarios, the object of interest may exhibit a lengthy strip-like structure, as exemplified by the GBM depicted in Figure 1. Using a large square pooled window does not capture bar information well. The strip pooling method can capture long dependencies more effectively [15].

Given a two-dimensional input tensor denoted as x, where x belongs to the space $R^{H\times W}$, H represents the spatial height, and W represents the spatial width. The size of the pooling window is d × d, and the size of the output two-dimensional tensor Y is $H_{0}\times W_{0}$. The standard pooling formula can be expressed as

(1) $$y_{i_{0},j_{0}}=\frac{1}{d\times d}\underset{0\leq i\leq d}{\sum }\underset{0\leq j\leq d}{\sum }x_{i_{0}\times d+i,j_{0}\times d+j}$$

where $0<i_{0}<{\;H}_{0},0<j_{0}<{\;W}_{0},H_{0}=H/d,$ and $W_{0}=W/d$.

It can be seen from Equation (1) that the standard pooling window is square, so it has an advantage in dividing square graphics. When the input image is striped, it cannot be segmented well.

The strip pooling method specifically solves the strip texture. Strip pooling differs from traditional two-dimensional average pooling in its unique window size, which is either (H, 1) or (1, W). Instead of averaging features across the entire two-dimensional plane, strip pooling focuses on averaging all features within a single row or column. Its expression is

(2) $$y_{i}=\frac{1}{W}\underset{0\leq j\leq W}{\sum }x_{i,j},y_{j}=\frac{1}{H}\underset{0\leq i\leq H}{\sum }x_{i,j}\;$$

where $y_{i}\;\in {\;R}^{H}$ and $y_{j}\;\in {\;R}^{W}$ respectively represent the strip pooling graph after strip pooling. Strip pooling enables the benchmark network to utilize long-distance dependencies effectively.

However, the strip pooling module (SPM) and the mixed pooling module (MPM) proposed in SPNet [15] have their own limitations. To effectively capture the varied influences posed by both long and non-long objects, we integrated both strip and pyramid pooling mechanisms into a composite mixed pooling module. This integration allows for a more comprehensive and nuanced treatment of feature extraction, catering to the distinct characteristics exhibited by diverse object shapes within the dataset. Therefore, we added both strip and pyramid pooling into a mixed pooling module, which considers the effects of long and non-long objects. However, the pyramid pooling operation is performed after the strip pooling operation, and there is no way to recover the information about the lost non-striped objects. The network is also very complicated. After carefully studying the entire network, the feature maps generated by the basic ResNet network at each stage are multiscale. In this paper, SSP operation was directly performed on the multiscale feature images f_3_, f_4_, and f_5_ generated by Res2Net, which not only makes the network model simpler but also takes into account the characteristics of strip and non-strip objects.

**Figure 3:**
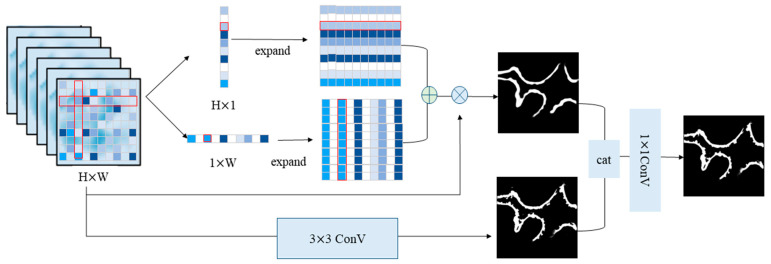
Schematic illustration of the refined strip pooling (RSP) module. This module incorporates both horizontal and vertical strip pooling methodologies to gather spatial context information along both the horizontal and vertical axes. It then connects the features obtained from the standard and strip pooling to obtain the final feature mapping. The red frames highlight the variation process of row or column vectors.

The RSP module is shown in Figure 3. This module employs horizontal and vertical strip pooling operations to gather contextual information along the spatial dimension, specifically capturing horizontal and vertical features. After obtaining the features from the standard pooling and strip pooling techniques, we integrated them to arrive at the ultimate feature mapping. The refined strip pooling (RSP) module emphasizes the consolidation of diverse contextual information via diverse pooling methods, thereby enhancing the distinguishing capabilities of its features.

In addition to the strip-like characteristics of the GBM, it can also be observed from Figure 1 that the textures of the middle and edges of the GBM are different. This also creates challenges for the precise extraction of the GBM. In Figure 2, the global map generated by our network offers a coarse approximation of the GBM’s location. To refine this, we employed a series of recurrent reverse attention modules that establish a correlation between regions and boundary cues, enabling a deeper exploration [23]. These attention modules were designed to dynamically learn from three parallel, sophisticated features. By applying reverse attention, our network is able to augment the boundary details of the GBM, resulting in a more precise localization.

Specifically, the feature denoted as the reversed attention output, R_i_, is derived through an element-wise multiplication (⊙) operation between the high-level output features, {f_i_ for i = 3, 4, 5}, and the corresponding reversed attention weights, R_i_. This computation is formalized in the following equation:

(3) $$R_{i}=f_{i}\;⊙\;A_{i}$$

The significance of the reverse attention weight, A_i_, lies in its pivotal role in facilitating the detection of prominent objects within a given context, and it can be formulated as follows:

(4) $$A_{i}=⊖\left(\sigma \left(S_{i+1}\right)\right)\;$$

where the sigmoid function is denoted by σ(·), and ⊖(·) represents a reverse operation that subtracts the input from a matrix E, where all entries are united. This erasing technique serves to enhance the prediction map, transforming crude and imprecise estimations into precise and comprehensive outcomes.

In Figure 2, the implementation of deep supervision is demonstrated for the three side outputs (namely, S1, S2, and S3) as well as the global map S0, indicated by the red dashed line. Each of these maps undergoes upsampling to ensure conformity with the dimensions of the ground truth map (GT). For each test, S3 achieved the best performance, so we chose S3 to compare with the GT image.

### 3.2. Loss Function and Deep Supervision

Due to the limited receptive field, low-level features retain an abundance of intricate details intertwined with background noise, which also have clear boundaries (critical to generating saliency maps). High-level features undergo numerous downsampling procedures, leading to a significant reduction in detailed information, but there are still consistent semantics and clear backgrounds. To focus more on difficult-to-classify samples in model training, self-adaptive loss is employed to diminish the influence of easily classifiable samples by decreasing their weights while simultaneously enhancing the emphasis on challenging-to-classify samples by increasing their respective weights. The loss function is defined as

(5) $$\mathrm{minL}=L_{\mathrm{IoU}}^{\omega }+L_{\mathrm{BCE}}^{\omega }$$

where $L_{\mathrm{IoU}}^{\omega }$ represents the weighted IoU. IoU is the result of dividing the overlapping part of the two regions, A and B, by the set part of the two regions and the global information of interest. In this paper, A is the original image and B is the predicted image.

(6) $$L_{\mathrm{IoU}}^{\omega }\left(A,B\right)=1-\frac{\sum_{i=1}^{H}\sum_{j=1}^{W}\left({\mathrm{gt}}_{\mathrm{ij}}{\;*\;p}_{\mathrm{ij}}\right){\;*\;\omega }_{\mathrm{ij}}}{\sum_{i=1}^{H}\sum_{j=1}^{W}\left({\mathrm{gt}}_{\mathrm{ij}}+p_{\mathrm{ij}}-{\mathrm{gt}}_{\mathrm{ij}}{\;*\;p}_{\mathrm{ij}}\right){\;*\;\omega }_{\mathrm{ij}}}$$

where $L_{\mathrm{BCE}}^{\omega }$ represents the weighted BCE [15], the cross-entropy calculation function in the two-class classification task, and the pixel-level loss function.

(7) $$L_{\mathrm{BCE}}^{\omega }=\frac{\sum_{i=1}^{H}\sum_{j=1}^{W}\omega_{\mathrm{ij}}\left({\mathrm{gt}}_{\mathrm{ij}}{\;*\;\mathrm{logp}}_{\mathrm{ij}}+(1-{\mathrm{gt}}_{\mathrm{ij}})\;*\;\log\left(1-p_{\mathrm{ij}}\right)\right)}{\sum_{i=1}^{H}\sum_{j=1}^{W}\omega_{\mathrm{ij}}}\;$$

where

$$\omega_{\mathrm{ij}}=1+{\gamma \alpha }_{\mathrm{ij}}$$

$$\alpha_{\mathrm{ij}}=\left|\frac{\sum_{m,n\in A_{\mathrm{ij}}^{1}}{\mathrm{gt}}_{\mathrm{mn}}}{\sum_{m,n\in A_{\mathrm{ij}}^{1}}1}-{\mathrm{gt}}_{\mathrm{ij}}\right|+\left|\frac{\sum_{m,n\in A_{\mathrm{ij}}^{2}}{\mathrm{gt}}_{\mathrm{mn}}}{\sum_{m,n\in A_{\mathrm{ij}}^{2}}1}-{\mathrm{gt}}_{\mathrm{ij}}\right|+\left|\frac{\sum_{m,n\in A_{ij}}{\mathrm{gt}}_{\mathrm{mn}}}{\sum_{m,n\in A_{ij}}1}-{\mathrm{gt}}_{\mathrm{ij}}\right|$$

where ${\mathrm{gt}}_{\mathrm{ij}}\in \left[0,1\right]$ represents the original image: if the pixel (i, j) is the GBM, ${\mathrm{gt}}_{\mathrm{ij}}=1$; otherwise ${\mathrm{gt}}_{\mathrm{ij}}=0$; $p_{\mathrm{ij}}\in \left[0,1\right]$ represents the probability that pixel (i, j) is the GBM; $\omega_{\mathrm{ij}}$ represents the weight value; ${\;\alpha }_{\mathrm{ij}}$ indicates the importance of pixels by calculating the difference between pixels and their surrounding pixels; $A_{ij}$, $A_{\mathrm{ij}}^{1},$ and $A_{\mathrm{ij}}^{2}$ represent the neighborhood of the pixel (i, j); and we used $A_{ij}$ as the 31 × 31 rectangle, $A_{\mathrm{ij}}^{1}$ as the 31 × 1 rectangle, and $A_{\mathrm{ij}}^{2}$ as the 1 × 31 rectangle. By using these rectangles, we can consider and locate the GBM like a doctor would by taking the tissues around the GBM as the reference object; γ is a super parameter, and the value is 5 in this paper.

To enhance the segmentation of the GBM, the SSPNet integrates strip pooling to capture the striped features of the GBM and a reverse attention mechanism to highlight edge information. Strip pooling, with window sizes of (H, 1) or (1, W), averages features along rows or columns, effectively capturing long-range dependencies. The reverse attention mechanism dynamically enhances boundary details, thereby improving segmentation accuracy. In the experimental section, work was carried out to boost model transparency and credibility. SSPNet employs feature map visualization to intuitively display how the model processes and extracts features. Quantitative analysis using metrics such as the Dice coefficient and IoU provided a clear understanding of the model’s segmentation accuracy. Comparative experiments with other advanced medical image segmentation methods demonstrated SSPNet’s superiority in segmenting the GBM. Integration with physical implications through quantitative data extraction, such as calculating the GBM thickness using skeleton extraction, enhanced the model’s practicality and decision-making credibility. These methods have ensured SSPNet’s effectiveness and interpretability in practical applications.

## 4. Experimental Design and Analysis

### 4.1. Experimental Details

**GBM Datasets and Baselines:** The experimental data used in this paper came from 700 electron microscopic images of renal biopsies, each with a resolution of 2048 × 2048 pixels, procured from 347 patients at the Southern Medical University. The dataset encompassed a diverse range of renal pathologies, including, but not limited to, primary chronic glomerular nephropathy (IgA nephropathy), minimal change disease (MCD), membranous nephropathy (MN), thin basement membrane nephropathy, diabetic nephropathy, and light mesangial proliferative glomerulonephritis.

To ensure the comprehensiveness and balance of the dataset, we analyzed the distribution of disease types and pathological variations across the 347 patients. The dataset included the following disease types and their respective sample counts:

- Primary chronic glomerular nephropathy (IgA Nephropathy): 74 patients (21.3%);
- Minimal change disease (MCD): 54 patients (15.6%);
- Membranous nephropathy (MN): 59 patients (17%);
- Thin basement membrane nephropathy: 26 patients (7.5%);
- Diabetic nephropathy: 33 patients (9.5%);
- Light mesangial proliferative glomerulonephritis: 53 patients (15.3%);
- Lupus nephritis: 48 patients (13.8%).

This distribution ensures that the dataset covered a wide spectrum of renal pathologies, minimizing potential bias toward any specific disease type. Additionally, the dataset included a variety of pathological variations within each disease category, such as different stages of glomerular basement membrane thickening, mesangial proliferation, and podocyte effacement. This diversity allowed the model to generalize well across different pathological conditions, and ensures robust performance in real-world clinical scenarios.

The original images were marked by the pathologist. Figure 1 shows an example of the dataset and annotation. The annotated information of the original data included compacts, foot processes, endothelial cells, mesangial area, and the GBM. This paper focused on extracting the thickness of the GBM, so only the annotated information regarding the GBM was selected. Within this information, there were no abnormal changes in the basement membrane of MCD, IgA, or mild mesangial proliferative glomerulonephritis.

Despite the vast amount of material available, there is little research on the semantic segmentation of the GBM. Therefore, in our research, we conducted a comparative analysis of our novel SSPNet approach against five state-of-the-art (SOTA) medical image segmentation methodologies, aiming to evaluate its performance and identify potential advantages. These other methodologies were Deeplabv3 [20], Deeplabv3+ [24], HarDNet-MSEG [23], F3Net [25], and PraNet [26].

**Training Settings and Metrics:** During the experimental phase, we randomly partitioned the dataset into three distinct subsets: a training set comprising 500 images, a validation set with 100 images, and a test set containing 100 images. Instead of utilizing data augmentation, we adopted a multiscale training approach with scales of 0.75, 1, and 1.25 to enhance model robustness.

For quantitative assessment, we adhered to the evaluation metrics employed in prior works [27,28], namely, the mean Dice coefficient and the mean Intersection over Union (IoU). To gain a more comprehensive understanding of our model’s performance, we additionally incorporated three supplementary metrics: S-measure (Sm), which assesses structural similarity between predictions and ground truths at both the regional and object levels [29]; weighted F-measure (Fm), a holistic performance indicator that balances weighted precision and recall [30]; mean absolute error (MAE), which quantifies the average pixel-wise difference between predicted and ground truth saliency maps [31].

**Implementation Details** The model in this paper was implemented under the Pytorch framework. We used six GPUs, which were NVIDIA GeForce RTX 3090 or NVIDIA TITANXp, sourced from NVIDIA Corporation (Santa Clara, CA, USA). The experimental operating system used was ubuntu14.04. To refine the optimization process, we adopted the Adam optimizer, tuning the overall parameters with a learning rate set at 3 × 10^−4^. The entire network underwent end-to-end training, utilizing a batch size of 2 across 60 epochs. All input data were consistently resized to 1024 × 1024 pixels to ensure uniformity. Subsequently, the performance of the proposed network architecture was benchmarked against alternative network configurations, facilitating a comprehensive comparative analysis.

To evaluate the real-time performance of our model, we measured the inference speed on a single NVIDIA GeForce RTX 3090 GPU. The average inference time per image was approximately 20 milliseconds (ms), which translates to a frame rate of approximately 50 frames per second (FPS). This high frame rate ensures that our model can process video streams in real time, making it suitable for practical applications such as automated pathological diagnosis in clinical settings.

### 4.2. Comparison of Pooling Operations

This paper compared the SSP model with the strip pooling module (SPM), the mixed pooling module (MPM), and the SPM + MPM models in SPNet. The experimental results are shown in Table 1. Our SSPNet surpassed the SPNet by a notable margin, achieving a mean Dice score improvement of over 1%. This observation underscores the model’s enhanced capacity for accurately segmenting intricate strip features, indicative of its superior learning proficiency. Therefore, better experimental results were obtained.

**Figure 4:**
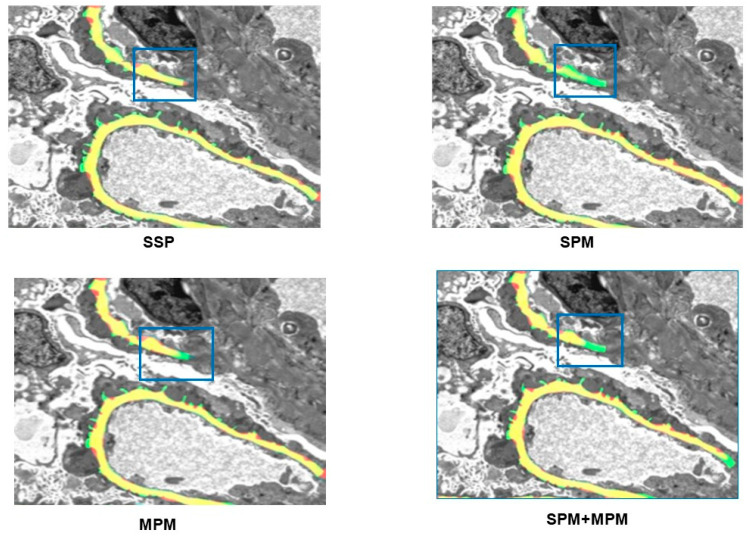
Qualitative results of the different methods. Green indicates professional labeling of nephropathy, red indicates automatic labeling by the algorithm, and yellow indicates overlapping parts. The blue boxes visually emphasize the notable differences in the outcomes produced by different methodologies.

In Figure 4, we provide the GBM segmentation results of SSPNet. Green indicates professional labeling of nephropathy, red indicates automatic labeling by the algorithm, and yellow indicates overlapping parts. It can be seen from Figure 4 that the strip information marked by SSP, especially the part framed by the blue box, is more comprehensive, and the difference is obvious.

### 4.3. Ablation Experiment

To verify the feasibility of each step in this method, ablation experiments were carried out in the specific optimization stage of this method to verify the correctness and necessity of each processing stage.

We first tested each module in isolation to verify its influence. As shown in Table 2, Res2Net was selected as the backbone of this paper. Res2Net + Dilated means Dilated was added to Res2Net. Res2Net + SSP refers to an SSP module being added to extract mixed textures for the basic network framework. Res2Net + AT indicates that an attention module was added to the basic network to enhance boundary information. Res2Net + LossA indicates that self-adaptive loss was used to replace the original loss. Additionally, we tested the overlapping influence by gradually adding the module. SSPNet is the proposed method.

It can be seen in Table 2 that the incorporation of dilated convolutions, facilitating exponential enlargement of the receptive field while maintaining resolution and coverage, resulted in an accuracy of 0.773, demonstrating an effective approach for the enhancement of performance. At the same time, the addition of an attention mechanism that emphasized boundary information also improved the performance of the final network. The accuracy was increased to 0.767. Then, in view of the strip texture features of the GBM, an SSP module was directly added on the basis of Res2Net to emphasize the extraction of strip texture features, and an accuracy rate of 0.756 was obtained. This shows that the SSP module has a good effect when processing images with striping problems. We changed the loss function and obtained an accuracy of 0.772. Finally, we gradually integrated the module, obtaining an accuracy of 0.792.

### 4.4. Performance Comparison

**Figure 5:**
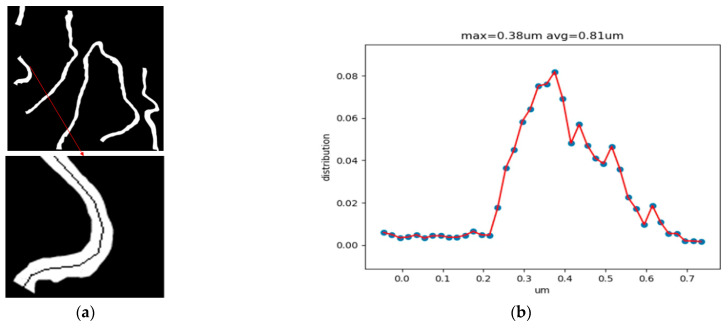
The thickness of the GBM was calculated using its skeleton GMB. (**a**) The skeleton of the GMB; (**b**) the thickness of the GBM. Red lines highlight magnified regions of key details, and blue dots mark the thickness measurement points of GMB.

It can be found in Table 3 that for strip images, such as those of the GBM, the SSP module can extract the strip texture, and the effect is better than that of other methods. Figure 5 shows the specific segmentation results. It can be intuitively observed that this method can predict the strip texture well. Finally, automatic thickness measurement of the GBM is helpful for pathologists in carrying out subsequent pathological diagnoses. We used the close morphological operation to remove some noise and then used the skeleton algorithm [29,30] to extract the skeleton of the GT graph. Taking the longest line segment in the skeleton as the central axis of the area, the distance from the point on the central axis to the background pixel was calculated and the thickness of the GBM obtained, providing quantitative information for doctors’ later diagnoses. We obtained digital features and drew a digital feature graph, which is shown in Figure 5.

To assess the statistical significance of the performance differences between SSPNet and other state-of-the-art models, we conducted paired *t*-tests on the Dice coefficients obtained from the test set. *p*-values and 95% confidence intervals (CIs) were calculated to quantify the significance of the differences (Table 4).

The statistical analysis revealed that SSPNet achieved significantly higher Dice coefficients compared to Deeplabv3, Deeplabv3+, HarDNet-MSEG, F3Net, and PraNet (*p*-values < 0.001 for Deeplabv3, Deeplabv3+, and HarDNet-MSEG; *p*-value = 0.012 for F3Net; *p*-value = 0.008 for PraNet). The 95% confidence intervals of the differences further support the robustness of SSPNet’s performance, indicating that the improvements are not due to random variation.

### 4.5. Experiments on Polyp Segmentation

In conclusion, the proposed model’s versatility was validated by testing on a publicly available polyp dataset, demonstrating its applicability beyond GBM detection tasks. To uphold a rigorous verification process, we adhered strictly to the training protocol stipulated in [26], where the Kvasir and CVC-ClinicDB (also known as CVC-612) image datasets were systematically partitioned, with 80% used for training, 10% for validation, and the remaining 10% for testing, and with all assignments being carried out randomly to ensure unbiased evaluation.

As evidenced by the results presented in Table 5 and Table 6, our methodology yielded favorable outcomes when applied to the polyp datasets, underscoring its robust performance and broad applicability in medical image semantic segmentation. Notably, our model, originally conceived for addressing strip structures, demonstrated its capability to deliver satisfactory results on generic datasets as well. This observation leads us to speculate that the employment of strip pooling in our approach facilitates the capture of long-range dependencies within the data, contributing to its enhanced performance and versatility.

### 4.6. Dataset Specificity and Generalizability

The dataset used in this study comprised 700 electron microscopic images of renal biopsies from 347 patients, covering a diverse range of renal pathologies, including IgA nephropathy, minimal change disease, and membranous nephropathy. The dataset’s diversity in terms of pathology types and variations ensured that SSPNet was trained and tested on a wide range of scenarios, which is crucial for robust performance. Additionally, the dataset’s balanced distribution of pathologies ensured that SSPNet learned generalized features rather than being overfit to specific disease types.

However, the images exhibited the following unique characteristics: high resolution (2048 × 2048 pixels) and low contrast, specifically between the GBM and surrounding tissues. Such attributes inherently influenced SSPNet’s performance. These characteristics may also have introduced limitations: models trained on high-resolution EM images might struggle with lower-resolution datasets, and dependency on edge attention could reduce effectiveness in high-contrast scenarios. While SSPNet demonstrated high precision in segmenting the GBM in this dataset, it is essential to evaluate its generalizability to other datasets. The observed improvements in Dice coefficients and IoU metrics were significant within the context of this study, but they may not be directly transferable to datasets with different imaging modalities, resolutions, or pathological conditions. For instance, datasets with higher contrast or different tissue structures might yield different performance metrics.

To evaluate generalizability, we tested SSPNet on the Kvasir and CVC-612 polyp segmentation datasets, which differ significantly in imaging modality, resolution, and target structures. As shown in Table 5 and Table 6, SSPNet achieved competitive Dice scores (0.908 and 0.915, respectively), outperforming established methods like PraNet. These results suggest that SSPNet’s design—particularly its strip pooling mechanism for capturing long-range dependencies and its adaptive loss function for emphasizing challenging regions—enhances its adaptability to diverse medical imaging tasks. However, the observed gains in GBM segmentation (Dice = 0.792) are partially context specific. For instance, the strip pooling module excels in segmenting elongated structures (e.g., the GBM), but may offer only marginal benefits for compact or irregularly shaped targets.

## 5. Conclusions

Accurate semantic segmentation of the glomerular basement membrane (GBM) and automatic measurements of its thickness are crucial for aiding pathologists in conducting follow-up pathological diagnoses. In this study, we proposed the self-adaptive strip pooling network (SSPNet) to address the challenges posed by the complex ultrastructure and irregular shape of the GBM. By leveraging the strip-like features of the GBM, SSPNet effectively extracted both strip and square textures through its innovative refined strip pooling (RSP) module. Additionally, the incorporation of a reverse attention mechanism significantly enhanced edge extraction, thereby improving segmentation accuracy. Through extensive experiments and comparisons with state-of-the-art methods, SSPNet demonstrated superior performance in GBM segmentation, achieving high precision and robustness.

**Figure 6:**
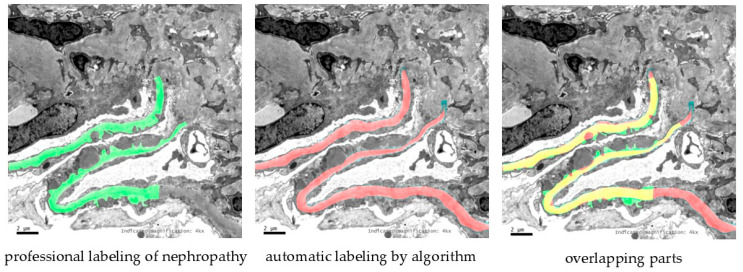
Green indicates professional labeling of nephropathy, red indicates automatic labeling by the algorithm, and yellow indicates overlapping parts.

However, despite its notable achievements, SSPNet has certain limitations. As shown in Figure 6, segmentation errors still occurred in some cases. One possible reason is the reliance solely on textural information without incorporating expert knowledge, which may be insufficient for accurate classification in all scenarios. Moreover, the manual annotations used for training and evaluation, performed by expert pathologists, are subject to inter-observer variability, a common issue in medical image analysis. This variability could subtly influence model training and evaluation results. Future studies should conduct inter-rater reliability analyses, such as using Cohen’s kappa or Dice agreement scores, across multiple annotators to quantify this effect. Furthermore, real-world clinical practices may involve differing diagnostic criteria or annotation granularity. To improve reproducibility, we recommend adopting consensus-driven annotation guidelines and integrating semi-automated tools to reduce human subjectivity [33]. These measures would strengthen SSPNet’s reliability and facilitate its application in diverse clinical workflows.

The proposed method’s performance on external polyp datasets (Table 5 and Table 6) suggests promising generalizability. However, its efficacy in real-world GBM segmentation depends on addressing the aforementioned challenges. For broader adoption, validation across multi-institutional cohorts and imaging modalities (e.g., optical microscopy or other EM protocols) is imperative. Collaborative efforts with clinical partners will be essential to refine SSPNet’s adaptability to practical diagnostic scenarios while ensuring ethical and regulatory compliance.

In the future, the following studies will be conducted:

(1) **Automated Data Annotation**: Investigation of methods for automated data annotation to improve the accuracy and efficiency of the training process. By developing algorithms that can automatically generate high-quality annotations, we aim to reduce the reliance on manual annotation and enhance the scalability of our approach.
(2) **Multi-class Segmentation and Expert Knowledge Integration**: Extend the segmentation framework to support multi-class segmentation tasks, enabling the simultaneous identification of multiple structures within renal biopsy images. Additionally, we will explore ways to incorporate expert knowledge and clinical guidelines into the segmentation process. By integrating expert experience and domain-specific rules, we aim to improve the accuracy and reliability of the segmentation results, making them more aligned with clinical needs.

## Figures and Tables

**Table 1:** Comparison of pooling operations.

Model	Dice↑	IoU↑	Sm↑	Fm↑	MAE↓
Original (Res2Net)	0.746	0.603	0.806	0.710	0.042
Res2Net + SPM	0.748	0.604	0.813	0.709	0.043
Res2Net + MPM	0.747	0.603	0.810	0.699	0.044
Res2Net + SPM + MPM	0.738	0.594	0.811	0.687	0.045
Res2Net + SSP	0.756	0.615	0.818	0.709	0.042

Note: ↑ indicates higher values are better, ↓ indicates lower values are better.

**Table 2:** Ablation study for SSPNet on the GBM dataset.

Model	Dice↑	IoU↑	Sm↑	Fm↑	MAE↓
Res2Net	0.746	0.603	0.806	0.710	0.042
+Dilated	0.773	0.637	0.812	0.742	0.038
+SSP	0.756	0.615	0.818	0.709	0.042
+AT	0.767	0.630	0.813	0.737	0.039
+LossA	0.772	0.637	0.818	0.716	0.041
+Dilated + SSP	0.776	0.642	0.821	0.730	0.039
+Dilated + SSP + AT	0.785	0.653	0.828	0.741	0.037
SSPNet	0.792	0.662	0.836	0.753	0.036

**Table 3:** Comparison of the results from the various models.

Model	Dice↑	IoU↑	Sm↑	Fm↑	MAE↓
Deeplabv3 [20]	0.760	0.625	0.819	0.730	0.040
Deeplabv3+ [24]	0.754	0.621	0.814	0.711	0.041
HarDNet-MSEG [23]	0.763	0.629	0.823	0.704	0.042
F3Net [25]	0.781	0.652	0.831	0.731	0.038
PraNet [26]	0.785	0.656	0.826	0.754	0.037
Our method	0.792	0.662	0.836	0.753	0.036

**Table 4:** Statistical significance analysis of the Dice coefficients.

Model	Mean Dice Coefficient	*p*-Value vs. SSPNet	95% CI of the Difference
Deeplabv3	0.760	<0.001	[0.012, 0.036]
Deeplabv3+	0.754	<0.001	[0.018, 0.042]
HarDNet-MSEG	0.763	<0.001	[0.015, 0.039]
F3Net	0.781	0.012	[0.003, 0.021]
PraNet	0.785	0.008	[0.002, 0.018]
SSPNet	0.792	-	-

Note: *p*-values indicate the significance of the differences between SSPNet and the other models. A *p*-value < 0.05 suggests a statistically significant difference. The 95% CIs of the differences show the range within which the true difference in the mean Dice coefficients is likely to fall.

**Table 5:** Comparison with the results of various models on Kvasir [28], with U-Net, U-Net++, ResU-Net, ResU-Net, and PraNet evaluation scores from [10]. Nan denotes that the results are unavailable.

Model	Dice↑	IoU↑	Sm↑	Fm↑	MAE↓
U-Net [20]	0.818	0.444	0.858	0.794	0.055
U-Net++ [24]	0.821	0.743	0.862	0.808	0.048
ResU-Net [23]	0.791	Nan	Nan	Nan	Nan
ResU-Net++ [25]	0.813	0.793	Nan	Nan	Nan
PraNet [26]	0.898	0.840	0.915	0.885	0.030
Ours (SASPNet)	0.908	0.859	0.924	0.909	0.018

**Table 6:** Comparison with the results of various models on CVC-612 [32], with U-Net, U-Net++, ResU-Net, ResU-Net, and PraNet evaluation scores from [10]. Nan denotes that the results are unavailable.

Model	Dice↑	IoU↑	Sm↑	Fm↑	MAE↓
U-Net [20]	0.823	0.755	0.954	0.889	0.019
U-Net++ [24]	0.794	0.729	0.931	0.873	0.022
ResU-Net [23]	0.779	Nan	Nan	Nan	Nan
ResU-Net++ [25]	0.796	0.796	Nan	Nan	Nan
PraNet [26]	0.899	0.849	0.979	0.896	0.009
Ours (SASPNet)	0.915	0.866	0.933	0.914	0.011

## Data Availability

The data presented in this study are available only upon request from the corresponding author, due to the fact that they are sourced from a third-party provider, whose terms of use and data-sharing policies strictly prohibit the unauthorized disclosure or public dissemination of the information. As the researcher utilizing these data, the author is bound by contractual obligations and ethical guidelines to respect the privacy and confidentiality agreements established by the data owner. Therefore, direct access to the raw data is limited to ensure compliance with these regulations and to maintain the integrity of the data-sharing framework. However, for purposes of verification, collaboration, or further analysis, the corresponding author is willing to facilitate requests for data access, subject to the approval of the third-party data provider and adherence to all relevant legal and ethical requirements.
